# Concomitant Cytotoxic Effector Differentiation of CD4^+^ and CD8^+^ T Cells in Response to EBV-Infected B Cells

**DOI:** 10.3390/cancers14174118

**Published:** 2022-08-25

**Authors:** Yumi Tamura, Keita Yamane, Yohei Kawano, Lars Bullinger, Tristan Wirtz, Timm Weber, Sandrine Sander, Shun Ohki, Yasuo Kitajima, Satoshi Okada, Klaus Rajewsky, Tomoharu Yasuda

**Affiliations:** 1 Department of Immunology, Graduate School of Biomedical and Health Sciences, Hiroshima University, Hiroshima 734-8551, Japan; 2 Department of Hematology, Oncology and Tumor Immunology, Chariteé-Universitätsmedizin Berlin, Corporate Member of Freie Universität Berlin, Humboldt-Universität zu Berlin, 13353 Berlin, Germany; 3 Immune Regulation and Cancer, Max-Delbrück-Center for Molecular Medicine in the Helmholtz Association (MDC), 13125 Berlin, Germany; 4 Department of Pediatrics, Graduate School of Biomedical and Health Sciences, Hiroshima University, Hiroshima 734-8551, Japan

**Keywords:** Epstein–Barr virus, LMP1, LMP2A, lymphoblastoid cell line, CD4^+^ CTL, T-bet, Eomes, Granzyme B, Perforin, CD107a

## Abstract

**Simple Summary:**

The Epstein–Barr virus (EBV) is a γ-herpes virus that primarily infects human B cells, and more than 90% of adults have experienced infection. EBV^+^ B cells express several viral proteins, transmitting signals important for the transformation and tumorigenesis of the infected B cells. Immune surveillance by the host immune system is important to suppress such abnormal expansion of EBV-infected B cells. Here we found that both CD4^+^ T cells and CD8^+^ T cells show similar gene expression patterns relating to cytotoxicity towards EBV-infected B cells. EBV-specific cytotoxic CD4^+^ T cells markedly expressed T-bet, Granzyme B, and Perforin alongside killing activity, which could reflect mechanisms shared with cytotoxic CD8^+^ T cells. Our findings support the concept that, upon EBV and perhaps other viral infections, T cells of different subsets can be drawn into common pathways mediating immune surveillance through cytotoxicity.

**Abstract:**

Most people infected by EBV acquire specific immunity, which then controls latent infection throughout their life. Immune surveillance of EBV-infected cells by cytotoxic CD4^+^ T cells has been recognized; however, the molecular mechanism of generating cytotoxic effector T cells of the CD4**^+^** subset remains poorly understood. Here we compared phenotypic features and the transcriptome of EBV-specific effector-memory CD4^+^ T cells and CD8^+^ T cells in mice and found that both T cell types show cytotoxicity and, to our surprise, widely similar gene expression patterns relating to cytotoxicity. Similar to cytotoxic CD8^+^ T cells, EBV-specific cytotoxic CD4^+^ T cells from human peripheral blood expressed T-bet, Granzyme B, and Perforin and upregulated the degranulation marker, CD107a, immediately after restimulation. Furthermore, T-bet expression in cytotoxic CD4^+^ T cells was highly correlated with Granzyme B and Perforin expression at the protein level. Thus, differentiation of EBV-specific cytotoxic CD4^+^ T cells is possibly controlled by mechanisms shared by cytotoxic CD8^+^ T cells. T-bet-mediated transcriptional regulation may explain the similarity of cytotoxic effector differentiation between CD4^+^ T cells and CD8^+^ T cells, implicating that this differentiation pathway may be directed by environmental input rather than T cell subset.

## 1. Introduction

The Epstein–Barr virus (EBV) is a γ-herpes virus that primarily infects human naïve B cells, and more than 90% of adult people have experienced infection [1]. Although most people infected by EBV in childhood usually present with either no or only mild symptoms and acquire specific immunity, they remain latently infected throughout their life [2]. EBV^+^ B cells express several viral proteins, including latent membrane protein 1 (LMP1) and 2A (LMP2A). These viral proteins transmit constitutive intracellular signals to promote cellular proliferation and play important roles in the transformation and tumorigenesis of the infected B cells [3]. Patients with primary immunodeficiencies, immunocompromised because of allograft transplantation or infection with the human immunodeficiency virus (HIV), often develop EBV-associated lymphoproliferative diseases and lymphomas [4]. Thus, immune surveillance by the host immune system is important to suppress the abnormal expansion of EBV^+^ cells and their malignancies.

Previous studies using mice expressing LMP1 specifically in B cells demonstrated that, like human EBV-infected cells, LMP1-expressing B cells are efficiently eliminated by T cells. Deleting immune surveillance from those mice resulted in rapid and fatal lymphoproliferation and lymphomagenesis, indicating a central role for LMP1 in the surveillance and transformation of EBV-infected B cells [5]. In this study, the superior role of CD4^+^ T cells compared to cytotoxic CD8^+^ T cells in the elimination of lymphoma cells was demonstrated [5]. Furthermore, LMP1 signaling leads to overexpression of cellular antigens including tumor-associated antigens. Presentation of those antigens on major histocompatibility complex class I (MHC-I) and II (MHC-II), together with the upregulation of the costimulatory ligands CD70, OX40L, and 4-1BBL, induces potent cytotoxic CD8^+^ T cells and CD4^+^ T cells, respectively [5,6]. These results suggest that EBV infection potentially induces EBV-related lymphoma-specific cytotoxic CD4^+^ T cells (CD4^+^ CTLs) and CD8^+^ T cells (CD8^+^ CTLs).

In humans, CD4^+^ CTLs rarely exist in circulating T cells; however, EBV-specific CD4^+^ CTLs were detected in the peripheral blood of patients who recovered from infectious mononucleosis [7]. Bulk and single-cell RNA sequencing of tumor-infiltrating lymphocytes revealed the existence of CD4^+^ T cells with cytolytic phenotype [8,9,10,11,12] and the presence of CD4^+^ T cells with direct killing activity against human tumor tissues [13], introducing CD4^+^ T cells as critical players in the regulation of anti-tumor immunity. T cell co-culturing with lymphoblastoid cell lines (LCL) from EBV-infected human peripheral blood B cells induces CD4^+^ CTLs and CD8^+^ CTLs, providing efficient adoptive cell immunotherapy for patients with EBV-associated malignancies such as Hodgkin’s lymphoma, nasopharyngeal carcinoma, and post-transplant lymphoproliferative disease (PTLD) [14], suggesting a possible role of EBV-induced CD4^+^ CTLs in controlling EBV-associated diseases. Although the major therapeutic source of cytolytic T cells are CD8^+^ T cells, the existence of anti-virus-specific CD4^+^ T cells has been known for a long time, though their therapeutic relevance in clinical settings has not yet been fully elucidated. Interestingly, EBV-induced CD4^+^ T cells have the potential to exhibit cytotoxicity against EBV-negative tumors in vitro, suggesting a strategy to use EBV-specific CD4^+^ T cells for anti-tumor immunotherapy [15,16].

In this study, we directly compared cellular phenotype, transcriptome, and functional molecules in EBV-induced CD4^+^ CTLs to CD8^+^ CTLs in mice and humans to explore the key properties of EBV-associated CD4^+^ CTLs.

## 2. Materials and Methods

### 2.1. Mouse Strains

Previously described Cγ1-Cre mice had been generated by targeting 129P2-derived embryonic stem (ES) cells and backcrossing to C57BL/6 mice [17]. Previously described Rosa26-LMP1^flSTOP^ [5] and Rosa26-LMP2A^flSTOP^ [18] had been generated by targeting C57BL/6-derived ES cells. Cγ1-Cre mice were crossed with Rosa26-LMP1^flSTOP^ and Rosa26-LMP2A^flSTOP^ mice to generate Cγ1-Cre–Rosa26LMP1^flSTOP^/Rosa26-LMP2A^flSTOP^ (GCB-LMP1/2) mice in which LMP1 and LMP2A are expressed in germinal center (GC) B cells. Animals were maintained in specific pathogen-free conditions and handled according to protocols approved by the LAGeSo, Berlin.

### 2.2. Preparation of Single-Cell Suspensions

Single-cell suspensions were prepared in Dulbecco’s modified Eagle’s medium (DMEM) (Gibco, Waltham, MA, USA or FUJIFILM Wako Chemicals, Osaka, Japan) supplemented with 1% heat-inactivated fetal calf serum (FCS) and 1 mM EDTA. To isolate mononuclear cells from the spleen, tissues were mashed using frosted glass microscope slides. Single cell suspension from the spleen was resuspended in Gey’s solution (130 mM NH_4_Cl, 5.0 mM KCl, 0.8 mM Na_2_HPO_4_, 0.18 mM KH_2_PO_4_, 5.6 mM Glucose, 0.03 mM Phenol red, 1.0 mM MgCl_2_, 0.3 mM MgSO_4_, 1.5 mM CaCl_2_, 13 mM NaHCO_3_) for red blood cell lysis. Cell debris was removed using a 40–100 µm nylon mesh cell strainer (CORNING, NY, USA).

### 2.3. In Vitro Mouse T Cell Culture

Splenic B cells were enriched by depletion of CD43^+^ cells with anti-CD43 microbeads (Miltenyi Biotech, Bergisch Gladbach, Germany) that reach 95–98% of B cell purity. MACS purified B cells or FACS purified T cells were cultured in DMEM supplemented with 10% FCS, 2 mM L-Glutamine, 1 mM Sodium pyruvate, 10 mM HEPES, 52 μM β-Mercaptoethanol, Non-essential amino acids, and Penicillin-streptomycin (10% FCS-DMEM). To establish LMP1/2A^+^ B lymphoma cell lines, MACS purified B cells from GCB-LMP1/2 mice spleen were stained for CD19 and CD3 surface markers, and CD19^+^CD3^-^ B cells were FACS sorted to eliminate T cells completely. Those B cells were intravenously transferred into Rag2^−/−^ or Rag2^−/−^ γC^−/−^ recipient mice to obtain lymphoma cell lines. For in vitro T cell culture, FACS purified CD4^+^ T or CD8^+^ T cells from spleen were co-cultured with γ-irradiated (3–4 Gy) LMP1/2A^+^ B lymphoma cells [19] at a 1:1–0.1 T cell to lymphoma cell ratio in 10% FCS-DMEM supplemented with 20 ng/mL mrIL-2 (Peprotech, Cranbury, NJ, USA).

### 2.4. Generation of Lymphoblastoid Cell Lines

Akata (+) cells of BL origin, kindly provided by Dr. Hironori Yoshiyama [20], were cultured in RPMI1640 medium (FUJIFILM Wako Chemicals) containing 10% FCS. The productive lytic replication of Akata (+) cells was induced by treatment with anti-human IgG [21]. All cells were maintained at 37 °C in a 5% CO_2_ humidified atmosphere. Blood was taken (with informed consent in accordance with the Ethical Committee for Epidemiology of Hiroshima University) from EBV-seropositive healthy donors. HLA typing was performed by Wakunaga Pharmaceutical. EBV-LCLs were produced by infecting peripheral blood mononuclear cells (PBMCs) with a culture supernatant of EBV-producing Akata (+) cells in the presence of cyclosporin A (CsA). PBMCs were plated at 5 × 10^6^ cells in a T25 culture flask in an upright position containing 2 mL of RPMI-1640 medium (FUJIFILM Wako Chemicals) supplemented with 10% FCS, 1% Penicillin-streptomycin, 200 ng/mL CsA, and 2 mL of EBV containing supernatant from Akata (+) cells. The obtained LCLs were maintained in RPMI-1640 medium supplemented with 10% FCS and 1% Penicillin-streptomycin.

### 2.5. In Vitro Human T Cell Culture

Blood samples were obtained from two EBV-seropositive healthy donors with informed consent under the approval of the Ethical Committee for Epidemiology of Hiroshima University. PBMCs were isolated from buffy coats with density centrifugation using lymphocyte separation medium 1077 (Takara Bio, Shiga, Japan). FACS-sorted CD4^+^ T cells or CD8^+^ T cells were plated at 1 × 10^5^ cells per well in a U-bottom 96-well plate and co-cultured with autologous LCL irradiated at 30 Gy starting at 40:1 responder to stimulator ratio in RPMI-1640 supplemented with 10% FCS, 1% Penicillin-streptomycin, and 10 ng/mL human recombinant IL-7 (BioLegend, San Diego, CA, USA). An amount of 10 ng/mL IL-2 was added from day 2 of co-culture. Cells were then re-stimulated with irradiated autologous LCL at a ratio of 1:1 responder to stimulator weekly for a total of 5 stimulation rounds. Thereafter, T cells were expanded in T25 culture flasks on a weekly basis counting, and fed/split as necessary to give a final density of 1–2 × 10^6^ T cells/cm^2^. All cell counts were given as viable cells via trypan blue exclusion of dead cells.

### 2.6. CD107a Assay

CD4^+^ T cells and CD8^+^ T cells co-cultured and re-stimulated with autologous LCL for 5 stimulation rounds were adjusted to 5 × 10^4^ T cells per well of U-bottom 96-well plates before the 6th re-stimulation. T cells were re-stimulated with 5 × 10^4^ autologous LCL and were spun down at 200 rpm for 2 min before incubation at 37 °C. Percentages of CD107a-expressing cells were measured by flow cytometry at 6, 12, 24, and 48 h after re-stimulation.

### 2.7. In Vitro Killing Assay

The in vitro killing assay was performed as described previously [5]. In brief, ex vivo expanded CD4^+^ T and CD8^+^ T cells were purified by FACS sorting. T cells were incubated with target cells at different effector to target ratios for 4 h in U-bottom 96-well plates, followed by intracellular active Caspase-3 staining (BD Biosciences, Franklin Lakes, NJ, USA). For MHC blocking, 10 μg/mL of anti-I-A/I-E (M5/114.15.2, BioLegend), anti-H-2Kb (AF6-88.5, BioLegend), and anti-H-2Db (KH95, BioLegend) blocking antibodies were used. In all killing assays, effector-target mixtures in U-bottom 96-well plates were spun at 200 rpm for 2 min before moving to the incubator, and cultures were stained for TCRβ, CD4, CD8a, hCD2, and CD19 to identify target and effector cells. Active Caspase-3 positive target cells represent apoptotic target cells. For human cell killing assay, target cells (LCL cells) were labelled with a CellTrace^TM^ Violet Cell Proliferation Kit (Invitrogen, Waltham, MA, USA) before use. T cells were co-cultured with 5 × 10^3^ target cells at effector/target ratios of 20:1, 10:1, 5:1, 2.5:1, and 1.25:1 for 4 h in U-bottom 96-well plates, followed by active Caspase-3 staining (Invitrogen). In all killing assays, the effector/target mixtures in U-bottom 96-well plates were spun down at 400 rpm for 2 min before incubation at 37 °C. Cultures were analyzed for active Caspase-3 levels in Cell Trace-labelled target cells. Percent-specific killing was calculated as follows; % specific killing = % apoptotic target cells in cultures with both effectors and targets—% apoptotic target cells in cultures with targets alone.

### 2.8. Flow Cytometry

Single-cell suspensions from mice were prepared in DMEM supplemented with 1% FCS and 1 mM EDTA, lysed red blood cells with Gey’s solution, and cell numbers were counted. After treatment with TruStain fcX antibody (BioLegend), cells were stained with the following antibodies coupled to FITC: phycoerythrin (PE), peridinin chlorophyll (PerCP)-Cy5.5, PE-Cy7, allophycocyanin (APC), Alexa647, Alexa700, Brilliant violet (BV) 421, BV510, BV605, BV650, BV711, or BV785, active Caspase-3, B220, CD3ε, CD4, CD8a, CD19, CD38, CD44, CD62L, CD138, Fas, γδTCR, GL-7, H2-K^b^, hCD2, I-A/I-E, IgD, IgM, NK1.1, PD-1, and TCRβ, purchased from Affymetrix eBioscience, BD Biosciences, or BioLegend. Samples were acquired on LSRFortessa (BD Biosciences) and analyzed using FlowJo software (v10.8.1, BD Biosciences). Cell sorting was performed on FACSAria (BD Biosciences). For human cells, all staining procedures were performed on ice or at 4 °C. Cells in single-cell suspensions were treated with TruStain fcX (clone 93, BioLegend) anti-human CD16/32 Fc receptor blocking antibodies to reduce non-specific labeling of the cells before staining. Single cells were stained with fluorochrome-labeled antibody cocktails for at least 15 min in the dark. The cells were acquired in FACS buffer (PBS containing 1% FCS, 1 mM EDTA, and 0.05% NaN_3_) supplemented with 1 μg/mL propidium iodide (PI) to exclude dead cells. For intracellular staining, cells were stained with Zombie Aqua (BioLegend) to exclude dead cells, and intracellular staining was performed using a Foxp3/Transcription Factor Staining Buffer Set (eBioscience, Waltham, MA, USA). All flow cytometric data were acquired on CytoFLEX S (Beckman Coulter, Brea, CA, USA) and analyzed using Flow Jo software. Monoclonal antibodies specific for human anti-CD4 (OKT4), anti-CD8a (HIT8a), anti-CD45RA (HI100), anti CD45RO (UCHL1), anti-CCR7 (G043H), anti-T-bet (4B10), anti-Perforin (B-D48), and anti-Granzyme B (GB11) were purchased from BioLegend. Anti-Eomes (WD1928) were purchased from eBioscience.

### 2.9. Microarray

Gene expression profiling was performed on FACS-sorted T cell samples using Affymetrix GeneChip Mouse Genome 430 2.0 Arrays, according to the manufacturer’s recommendations (Affymetrix, Santa Clara, CA, USA) and data analysis was performed as previously reported [22,23]. Fluorescence ratios were normalized by applying the RMA algorithm using the BRB Array Tools software package (available at https://brb.nci.nih.gov/BRB-ArrayTools/, accessed on 26 April 2016). For subsequent analyses, we only included probe sets whose expression varied, as previously described (genes with a log intensity variation of *p*-value > 0.01 were excluded) [22]. Normalized gene expression data were then mean-centered and gene expression changes were shown as log2 fold change. These data were then interpreted in comparison to gene expression results obtained in naïve T cells. The complete microarray data are available at the Gene Expression Omnibus (http://www.ncbi.nlm.nih.gov/projects/geo; accession number GSE206802). For global gene expression analysis, genes that showed 2-fold or greater changes on either CD4^+^ T_EM_ (5380 genes) or CD8^+^ T_EM_ (7019 genes) compared to naïve T cells were selected. These genes were combined and duplicates eliminated; finally, 8323 differentially regulated genes were obtained. Scatter plots were generated using the mean values, and correlation coefficients were calculated by R.

### 2.10. Identification of Genes Related to Cytotoxicity in Mouse CD4^+^ CTLs

To obtain a gene list related to cytotoxic activity, GO:0001913 T cell-mediated cytotoxicity, GO:0001779 natural killer differentiation, GO:0019835 cytolysis, GO:0042267 natural killer cell-mediated cytotoxicity, and GO:0045065 cytotoxic T cell differentiation were selected as the relevant terms. After the elimination of duplicated genes and 4 genes being annotated as negative regulators of cell cytotoxicity, 194 genes were finally obtained. Next, 2839 genes that upregulated more than 2-fold upon CD4^+^ T cell differentiation from naïve to effector T cells were obtained. Then, 40 genes shared with the 194 and 2839 genes as gene sets associated with the cytotoxicity of LMP1/2A-induced CD4^+^ CTLs in mice were extracted.

### 2.11. Statistical Analysis

Prism software (GraphPad, San Diego, CA, USA) version 9.0 was used to perform two-way ANOVA with Tukey post-hoc tests. Asterisks indicate statistical significance for *p*-values < 0.05 (single), < 0.01 (double), < 0.001 (triple), and < 0.0001 (quadruple).

## 3. Results

### 3.1. Growth and Differentiation of T Cells Co-Cultured with B Cell Lymphoma Cells Expressing LMP1 and LMP2A

**Figure 1:**
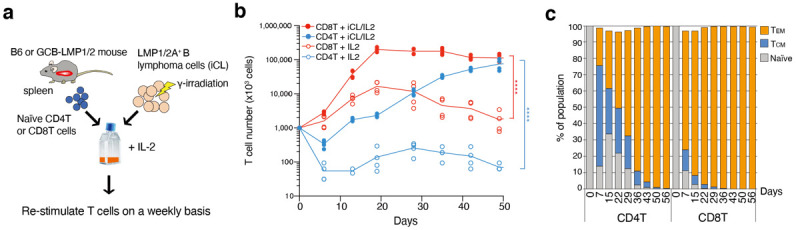
LMP1/2A^+^ B lymphoma cells induce T cell activation, expansion, and effector differentiation. (**a**) Workflow of in vitro LMP1/2A^+^ B cell-specific T cell culture. (**b**) Cell number of in vitro cultured CD4^+^ T cells or CD8^+^ T cells from C57BL/6 (B6) mice in the presence or absence of γ-irradiated LMP1/2A^+^ B lymphoma cells (iCL). Results from two to four biological replicates in each condition are shown. Statistical significance was tested between the presence and absence of iCL conditions (n = 4). Two-way ANOVA with Tukey post-hoc tests; **** *p* < 0.0001. (**c**) Percentage of T cells with CD62L^+^ CD44^-^ naïve, CD62L^-^ CD44^+^ effector-memory (T_EM_), and CD62L^+^ CD44^+^ central-memory (T_CM_) phenotypes on indicated time points. Naïve T cells were FACS sorted on day 0 from B6 mice (n = 2). Representative data of more than two independent experiments are shown. (**b**,**c**).

Most human EBV-associated B-cell lymphomas arise from GC B cells, which usually express both LMP1 and LMP2A. To characterize specifically activated cytotoxic effector T cells against EBV-related lymphoma cells, we used a genetic mouse model in which GC B cells express LMP1 and LMP2A [18]. These mice were generated by crossing Rosa26-LMP1^flSTOP^ and Rosa26-LMP2A^flSTOP^ mice with GC B cell-specific Cγ1-Cre mice (GCB-LMP1/2 mice hereafter). While there was no lymphoma observed in GCB-LMP1/2 mice at the immune-competent state, the transfer of purified B cells after the elimination of interacting T cells from GCB-LMP1/2 mouse spleen of these mice into the immunocompromised Rag2-deficient mice resulted in LMP1/2A^+^ B lymphoma development [19]. These findings indicate that T cell-mediated immune surveillance is established in GCB-LMP1/2 mice and persistently suppresses lymphoma development. We used established LMP1/2A^+^ B lymphoma cell lines as a stimulator to analyze T cells participating in immune surveillance in GCB-LMP1/2 mice. Naïve CD4^+^ T cells (TCRβ^+^ CD4^+^ CD62L^+^ CD44^−^) and CD8^+^ T cells (TCRβ^+^ CD8^+^ CD62L^+^ CD44^−^) were two-way sorted from stained single-cell suspensions of wild-type C57BL/6 mouse spleen, and those T cells were then independently co-cultured with γ-irradiated LMP1/2A^+^ B lymphoma cells in the presence of a low dose of IL-2. During the culture, T cells were re-stimulated with γ-irradiated LMP1/2A^+^ B lymphoma cell line (iCL) every week (Figure 1a). Under our culture conditions, both CD4^+^ T cells and CD8^+^ T cells showed vigorous cell expansion dependent on the iCL stimulator (Figure 1b). CD8^+^ T cells expanded much faster than CD4^+^ T cells and reached 200-fold higher cell numbers over input after 20 days. In contrast, CD4^+^ T cells expanded relatively slowly but continuously for 50 days of culture and reached about 100-fold higher cell numbers over input. FACS analysis of the naive/effector/memory phenotype in each T cell subset showed that the ratio of effector-memory phenotype dramatically increased in the culture period, indicating that co-culture with LMP1/2A^+^ B lymphoma cells induces differentiation of both naïve CD4^+^ T cells and CD8^+^ T cells into effector T cells (Figure 1c). As expected from the higher cell expansion of CD8^+^ compared to CD4^+^ T cells during the initial 20 days, CD8^+^ T cells increased the ratio of the effector-memory phenotype much faster than CD4^+^ T cells. In both CD4^+^ T cells and CD8^+^ T cells, the percentages of effector-memory phenotype reached almost 100% by 50 days and 30 days, respectively.

### 3.2. Effector CD4^+^ T Cells Induced by LMP1/2A^+^ B Lymphoma Cells Show MHC Class Ⅱ-Dependent Cytotoxicity

**Figure 2:**
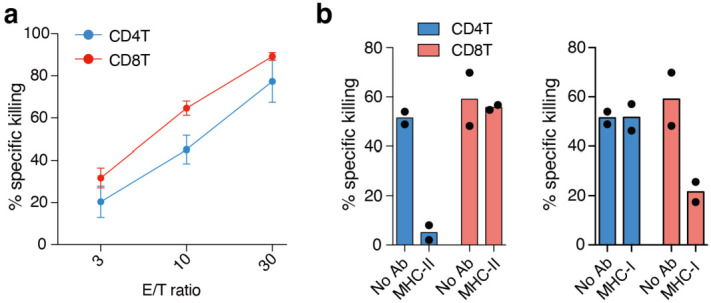
Activated CD4^+^ T cells and CD8^+^ T cells showed MHC-dependent cytotoxicity against lymphoma cells. (**a**) Naïve CD4^+^ T cells and CD8^+^ T cells were independently co-cultured with iCL for 24–31 days, as shown in Figure 1a. T cells were isolated and tested for killing activity by indicated E/T ratios. Mean +/− SD from two to eight biological replicates are shown on both CD4^+^ and CD8^+^ T cells. (**b**) In vitro-expanded CD4^+^ and CD8^+^ T cells from GCB-LMP1/2 mice were tested for MHC-dependency of killing activity at 30 E/T ratios in the presence or absence of MHC-I (H2K/H2D) or MHC-II (I-A/I-E) blocking antibodies (n = 2). (**a**,**b**) Representative data of more than two independent experiments are shown.

In the previous study, MHC-dependent cytotoxicity of both effector CD4^+^ T and CD8^+^ T cells activated by LMP1-expressing B lymphoma cells was shown against those lymphoma cells [5]. Therefore, we first examined the killing activity of naïve T cells against LMP1/2A^+^ B lymphoma cells. When naïve CD4^+^ T cells and CD8^+^ T cells separately co-cultured with LMP1/2A^+^ B lymphoma cells for 24 days, both CD4^+^ T cells and CD8^+^ T cells exhibited cytotoxicity against those LMP1/2A^+^ lymphoma cells (Figure 2a). Next, we examined whether in vitro-expanded CD4⁺ T and CD8⁺ T cells stimulated with iCL exhibit cytotoxic activity against the lymphoma cells. To examine whether those T cells recognize and show specific killing against lymphoma cells in an MHC dependent manner, we blocked MHC class I and II with the specific antibodies in the cytotoxic killing assay. We found that CD8^+^ T cells showed decreased cytotoxic activity, specifically upon MHC-I blockade, whereas MHC-II antibody treatment did not affect their killing activity. In contrast, CD4^+^ T cells showed cytotoxic activity dependent on MHC-II but not MHC-I (Figure 2b). Based on these results, we conclude that the effector CD4^+^ T cells activated by LMP1/2A^+^ B lymphoma cells contain CD4^+^ CTLs that recognize and kill lymphoma cells in an MHC-II-specific manner, while CD8^+^ CTLs kill target cells dependent on MHC-I as expected.

### 3.3. CD4^+^ and CD8^+^ CTLs Induced by LMP1/2^+^ Lymphoma Cells Show Highly Similar Gene Expression Changes

**Figure 3:**
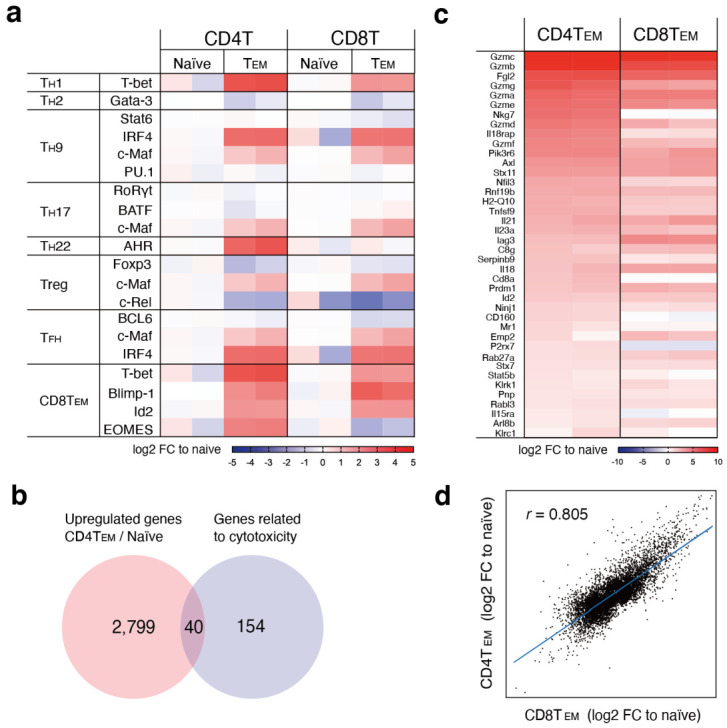
Characterization of LMP1/2A^+^ B lymphoma cell-specific T cells. Both CD4^+^ T_EM_ cells and CD8^+^ T_EM_ cells were purified on day 25 of in vitro cell culture with iCL and analyzed for RNA expression by microarray. Expression values are normalized, and log2 fold-change (FC) values are calculated to each naïve T cell (n = 2). (**a**) Expression pattern of master regulators of various T cell subset differentiation programs. (**b**) The Venn diagram depicts the number of genes upregulated in CD4^+^ T_EM_ cells compared to naïve CD4^+^ T cells and the number of genes associated with cytotoxicity based on GO terms. (**c**) The heat map shows log2 FC values of 40 genes overlapped in (**b**). (**d**) The scatter plot shows the mean log2 FC values of global gene expression in CD4⁺ T_EM_ cells (y-axis) and CD8⁺ T_EM_ cells (x-axis) compared to naïve T cells. The correlation coefficient is shown.

We characterized LMP1/2A^+^ B cell-specific CD4^+^ T cells by comparing their transcriptomes with those of effector-memory T (T_EM_) cells and naïve T cells. We isolated RNA from CD4^+^ T cells and CD8^+^ T cells of both naïve and T_EM_ and performed microarray analysis. We compared the expression change of known transcriptional regulators related to T cell subset differentiation between naïve T cells and T_EM_ cells and found that, surprisingly, the overall pattern of gene expression change was very similar between CD4^+^ T_EM_ cells and CD8^+^ T_EM_ cells (Figure 3a). CD4^+^ T_EM_ cells showed the induction of multiple genes, such as *Tbx21*, *IRF4*, *MAF*, *AHR*, *PRDM1*, *Id2*, and *Eomes*, which are involved in the differentiation of diverse T cell subsets. Of note, many CD8^+^ T_EM_ genes, *Tbx21*, *PRDM1*, and *Id2* were upregulated in CD4^+^ T_EM_ cells, indicating that EBV-induced CD4^+^ CTLs and CD8^+^ CTLs share common transcriptional regulators upon differentiation.

Next, to characterize the cytotoxic activity of CD4^+^ CTLs, we analyzed the expression of genes involved in cytotoxicity in CD4^+^ T_EM_ cells. One hundred ninety-four genes that promote cell cytotoxic activity according to their gene ontology (GO) terms were intersected by the 2839 genes, which are upregulated upon CD4^+^ T cell differentiation from naïve to T_EM_, to finally define 40 genes related to cytotoxicity (Figure 3b). These 40 genes were ranked according to their fold expression changes compared to naïve T cells. The top-ranked genes were enriched for molecules that are directly involved in the cell killing, such as *granzymes* and *NKG7*. Granzymes are the key killing molecules released from cytoplasmic granules of cytotoxic T cells and natural killer cells [24]. NKG7 is an NK cell granule protein that regulates cytotoxic granule exocytosis and inflammation [25]. The expression change of these 40 genes was compared between CD4^+^ T_EM_ cells and CD8^+^ T_EM_ cells, and we found that 80% of genes (32/40) are similarly upregulated in both CD4^+^ and CD8^+^ T_EM_ cells (Figure 3c). Furthermore, the expression change of global transcripts showed a notable similarity and correlation between these two cell types, indicating that CD4^+^ T_EM_ cells are similar to CD8^+^ T_EM_ cells, not only with respect to cytotoxicity but also in overall transcriptional regulation (Figure 3d). Thus, the CD4^+^ T_EM_ cells activated by LMP1/2A^+^ B lymphoma cells are similar to CD8^+^ CTLs in terms of transcriptional regulation, supporting the idea of concomitant activation of EBV-induced CD4^+^ CTLs with CD8^+^ CTLs in this experimental setting.

### 3.4. Proliferation and T_EM_ Cell Differentiation from Human Peripheral Blood Cells Responding to EBV^+^ B Cells

**Figure 4:**
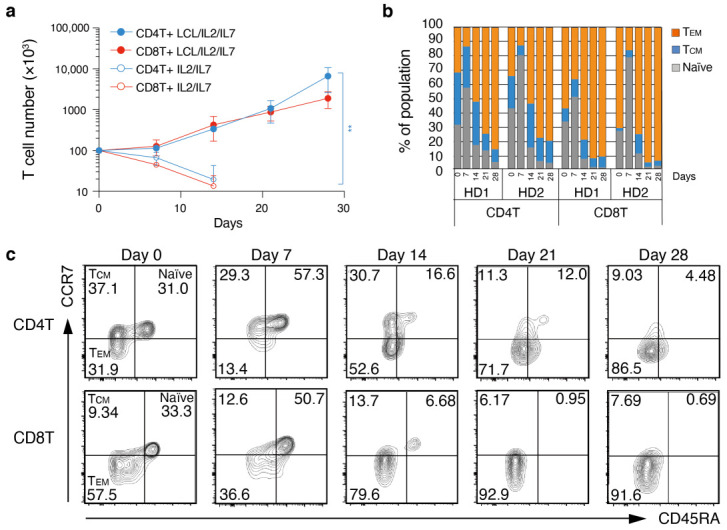
Long-term in vitro expansion and phenotypic analysis of LCL-specific human CD4^+^ T cells and CD8^+^ T cells. (**a**) Cell number of CD4^+^ T cells and CD8^+^ T cells from two healthy donors (HD1 and HD2) at each time point of co-culture with or without autologous LCL in the presence of 10 ng/mL IL-2 and 10 ng/mL IL-7. Results from four biological replicates in each condition are shown (n = 4). Two-way ANOVA with Tukey post-hoc tests; ** *p* < 0.01. (**b**) Percentage of CCR7^+^CD45RA^+^ naïve, CCR7^-^ CD45RA^-^ effector memory and CCR7^+^CD45RA^-^central-memory immune phenotype of LCL-stimulated T cells from HD1 and HD2 at the indicated time points (n = 2). (**c**) Representative contour plot of CCR7 and CD45RA expression on CD4^+^ T cells (upper panels) or CD8^+^ T cells (lower panels) at the indicated time points. HD1 is shown. (**a**–**c**) Representative data of more than two independent experiments are shown.

Next, we aimed to characterize EBV-specific human T cells. Because lymphoblastoid cell lines (hereafter LCL) are widely used to induce EBV-specific effector T cells, we first established LCLs by infecting in vitro cultured human peripheral blood B cells with EBV prepared from Akata (+) cells, of Burkitt lymphoma origin. Then, FACS-purified human peripheral blood CD4^+^ T cells and CD8^+^ T cells were co-cultured with γ-irradiated autologous LCLs in the presence of IL-2 and IL-7, and T cells were stimulated with LCL every week. We used IL-7 in a combination with IL-2 in this experiment because it was reported that putative CD4^+^ CTL precursors express high levels of the IL-7 receptor and undergo significant clonal expansion [26,27]. The number of both CD4^+^ T cells and CD8^+^ T cells increased over time upon co-cultivation with LCL, but failed to expand in their absence (Figure 4a). We also performed a flow cytometric analysis to determine the effector phenotype of T cells under co-culture conditions and noticed that the percentage of CCR7^-^ CD45RA^-^ effector T cells increased over time for both CD4⁺ T cells and CD8⁺ T cells, while the percentage of CCR7⁺ CD45RA⁺ naïve T cells decreased (Figure 4b,c). In CD8^+^ T cells, the ratio of effector-memory phenotype increased more than in CD4^+^ T cells. Although these observations were similar to our results in mice, the experimental workflow precluded a direct comparison of mouse and human data; in the animal experiments, naïve T cells were purified for the in vitro cultures, whereas the T cells in the human assays included memory T cells. Nevertheless, the results clearly indicate that T cells obtained from human peripheral blood became activated, expanded, and differentiated to effector T cells efficiently in the presence of LCLs.

### 3.5. Human EBV^+^ B Cell-Specific Cytotoxic CD4^+^ T Cells and CD8^+^ T Cells Are Induced by Co-Culture with LCLs

**Figure 5:**
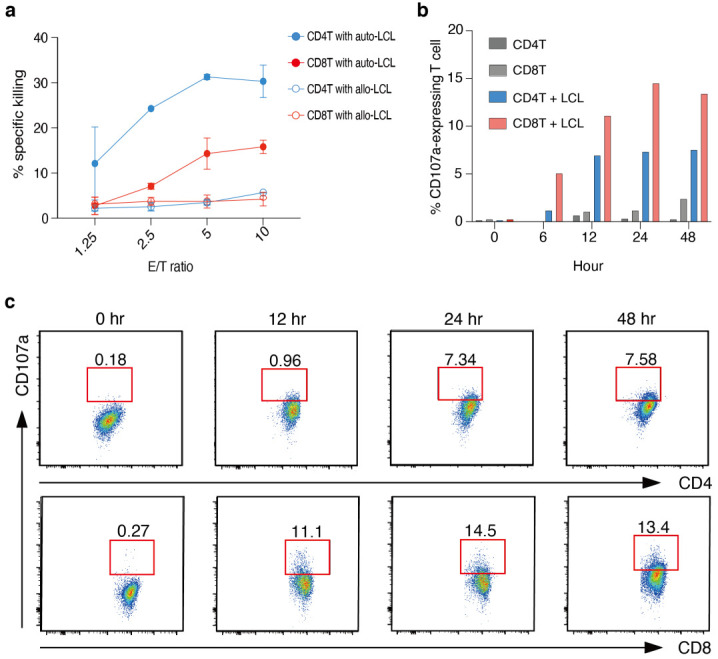
Cytotoxic activity of LCL-specific human CD4^+^ and CD8^+^ T cells expanded in culture. CD4^+^ T cells and CD8^+^ T cells from HD1 were co-cultured independently for more than 16 days with autologous LCL and assessed with the following experiments. Representative data of more than two independent experiments are shown. (**a**) Cytotoxic activity of LCL-specific CD4^+^ T cells and CD8^+^ T cells against autologous LCL or HLA-mismatched LCL at effector to target ratios of 10:1, 5:1, 2.5:1, and 1.25:1. The Mean +/- SEM is depicted for CD4^+^ and CD8^+^ T cells on day16 post-culture (n = 2). (**b**) The percentages of CD107a-expressing CD4^+^ T cells and CD8^+^ T cells at the indicated time points after re-stimulation with autologous LCL or without re-stimulation. (**c**) Representative profiles of CD107a expression in CD4^+^ CTL (upper panels) and CD8^+^ CTL (lower panels) at the indicated time points from re-stimulation. The gate delineates CD107a-positive cells.

A previous study reported that LCL-activated effector T cells from both CD4^+^ T and CD8^+^ T cells show cytotoxic activity against autologous LCLs [16]. Therefore, we performed in vitro killing assays to determine whether human T cells stimulated by LCLs show comparable cytotoxic activity in our culture condition. In agreement with the published data [16], we observed killing activity against autologous, but not allogenic LCLs for both CD4^+^ T and CD8^+^ T cells co-cultured for 16 days with EBV-infected B cells (Figure 5a). This suggests that the killing activity of both CD4^+^ CTLs and CD8^+^ CTLs is MHC restricted. In addition, we reproducibly observed higher killing activity in CD4^+^ CTLs compared to CD8^+^ CTLs. Next, to learn how these T cells killed target cells, we analyzed CD107a expression, a surface marker required for the degranulation of cytotoxic molecules [28], on the surface of CD4^+^ T cells and CD8^+^ T cells by flow cytometry. Not only CD8^+^ T cells but also CD4^+^ T cells showed up-regulation of CD107a on their cell surface immediately after the re-stimulation with the target cells (Figure 5b,c). These results suggest that LCL-induced CD4^+^ CTLs may kill target cells through the release of cytotoxic granules such as CD8^+^ CTLs. Thus, we conclude that in vitro culture of human CD4^+^ T cells in the presence of EBV^+^ B cells efficiently induces the differentiation of CD4^+^ CTLs, which have overlapping cytotoxic features with CD8^+^ CTLs, and potentially kill virally infected cells by the same pathways.

### 3.6. Co-Expression of T-Bet and Granzyme/Perforin Molecules in Human CD4^+^ CTL

Finally, we examined key regulatory molecules related to the effector function of EBV-specific CD4^+^ CTLs. From transcriptome analysis of CD4^+^ CTL using the mouse LMP1/2A transgenic system, we identified several key molecules specifically upregulated in mouse CD4^+^ CTLs, such as T-bet, Eomes, and Granzymes. The T-box family transcription factors, T-bet, and Eomes have been known as the master regulators of cytotoxicity in CD8^+^ CTLs [29,30]. While T-bet is a master regulator of Th1 lineage and IFN-γ production, recent studies have shown that it binds to the promoter of *GzmB* and *Prf1* and is functionally important for the expression of *GzmB* in CD4^+^ CTLs during influenza virus infection in mice [31]. Eomes is also expressed in cytotoxic CD4^+^ T cells capable of producing Granzyme B in a mouse chronic EAE model [32]. However, the expression kinetics of T-bet and Eomes in human CD4^+^ CTLs and the correlation of these transcription factors with the expression of Granzyme B and Perforin are not well known.

**Figure 6:**
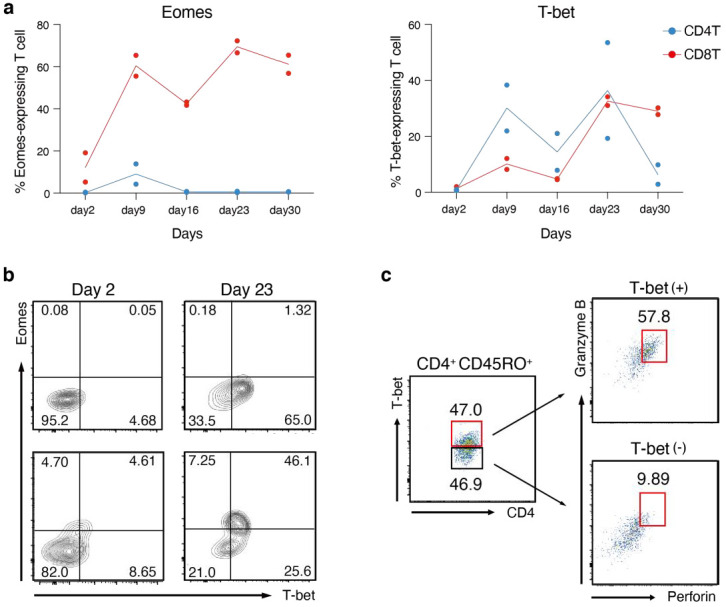
LCL-specific human CD4^+^ CTLs are characterized by low Eomes and high T-bet expression. (**a**) The percentages of Eomes-expressing (left graph) or T-bet-expressing (right graph) CD8⁺ T cells (in red) and CD4^+^ T cells (in blue) isolated from 2 healthy donors and co-cultured for the indicated time points with LCL (n = 2). (**b**) Representative contour plot of Eomes and T-bet expression of CD4^+^ T cells (upper panels) and CD8^+^ T cells (lower panels) on day 2 and day 23 post-culture from HD1. (**c**) Dot plot showing the gradient T-bet expression in CD4^+^ CD45RO^+^ T cells at day 16 post-culture (left panel). Granzyme B and Perforin expression in T-bet^high^ (right upper panel) and T-bet^low^ (right lower panel) cell populations are shown. The gate delineates double-positive cells. (**a**–**c**) Representative data of more than two independent experiments are shown.

Therefore, we analyzed the protein expression of T-bet and Eomes in the course of human T cell stimulation with LCL cells. CD8^+^ T cells were characterized by stable and strong T-bet and Eomes expression during the culture period. Whereas CD4^+^ T_EM_ cells highly expressed both Eomes and T-bet in mice, human CD4^+^ T cells expressed Eomes only in around 10% of cells on day 9, and we could not detect Eomes protein on day 16 and at later time points. In contrast, we could detect T-bet-expressing CD4^+^ CTLs from day 9 to day 30 at an equivalent or even higher frequency compared to CD8^+^ CTLs (Figure 6a,b). Therefore, we thought that T-bet expression may correlate with the expression of cytotoxic effector molecules, Granzyme B, and Perforin. Effector CD4^+^ T cells were gated to divide them into T-bet^high^ and T-bet^low^ populations. While T-bet^low^ CD4^+^ T cells barely expressed Granzyme B and Perforin, T-bet^high^ cells highly expressed both proteins (Figure 6c). Thus, we conclude that the expression of T-bet, but not Eomes, strongly correlates with the cytotoxic properties of EBV-induced human CD4^+^ CTLs, and that this may contribute to the similarities of cytotoxic function and effector cell differentiation between CD4^+^ T_EM_ and CD8^+^ T_EM_ cells in humans and mice.

## 4. Discussion

Accumulated evidence has revealed that CD4^+^ CTLs play an important role in various pathologic conditions such as infectious diseases, autoimmune diseases, and cancers [33]. The present study demonstrates that CD4^+^ and CD8^+^ CTLs induced by LMP1 and LMP2A-expressing B lymphoma cells in a mouse model show similar characteristics in terms of cytotoxic activity, T_EM_ differentiation, and the underlying gene expression programs. The microarray analysis of CD4^+^ CTLs and CD8^+^ CTLs induced by irradiated LMP1/2A^+^ B lymphoma cells revealed that both cell types highly upregulate transcription factor genes that are known to be critical for the function of CD8^+^ effector-memory CTLs such as *Tbx21*, *PRDM1*, and *Id2*. In addition, to our surprise, the overall expression pattern of T cell transcriptional regulators (Figure 3a) or even global genes (Figure 3d) revealed striking similarities between CD4^+^ and CD8^+^ T_EM_ cells, suggesting shared molecular mechanisms regulating differentiation of functionally similar T cell subsets. It has been reported that genes related to cytotoxicity, such as *Granzyme*s, *NKG7*, and *IL18RAP*, are upregulated in CD4^+^ CTLs in a mouse viral infection model, similar to what we saw in the present study, suggesting that these genes are generally induced in CD4^+^ CTLs upon viral infections [34]. In humans, CD4^+^ CTLs from infectious mononucleosis patients or CMV-infected individuals have been shown to express *Tbx21*, *Granzymes*, and *NKG7*, as we have demonstrated [7,35].

In our study, the transcription factor Eomes was markedly upregulated at the protein level in CD8^+^ CTLs, but minimal expression was observed in human LCL-induced CD4^+^ CTLs. Eomes and T-bet are related to T-box transcription factors regulating T cell development. In CD8^+^ T cells, Eomes is known to complement T-bet function and is essential for the acquisition of cytotoxicity [29]. Furthermore, Eomes is highly expressed upon prolonged TCR stimulation in combination with IL-2 [36]. In a recent report, cytomegalovirus-reactive CD4^+^ CTLs were detected in human peripheral blood in which the abrogation of Eomes did not affect Granzyme B and Perforin expression, whereas T-bet knockdown resulted in the downregulation of both effector molecules [37]. This study indicates that T-bet, but not Eomes, is essential for the acquisition of cytotoxicity in CD4^+^ T cells. However, in our mouse model, CD4^+^ CTLs upregulated Eomes expression. In similar studies, mouse CD4^+^ CTLs induced by LMP1-expressing lymphoma cells revealed high Eomes expression whose knockout diminished the cytotoxicity of the CD4^+^ T cells [6,15]. These seemingly inconsistent reports may be due to multiple mechanisms of CD4^+^ CTL differentiation regulated by diverse stimulators and environmental conditions. The conditions leading to Eomes induction and its significance in CD4^+^ T cells remain to be further assessed.

We and others have previously demonstrated that LMP1 expression in mouse and human B cells up-regulates MHC-I and -II molecules on the cell surface and enhances antigen presentation [5,38,39]. In contrast, LMP2A has been shown to suppress MHC-II expression by regulating E47 and PU.1 in B cells [40]. We also reported that LMP2A- and LMP1/LMP2A-expressing GC B cells express MHC-II at a low level in mice. Nevertheless, LMP1/LMP2A-expressing B cells were killed by CD4^+^ CTLs in an MHC-II-dependent manner, indicating that the expression level of MHC-II is sufficient to induce the effector function of CD4^+^ CTLs [18]. Therefore, the LMP1 signal in mouse and human B cells could counteract LMP2A to sustain MHC-II expression for T cell recognition. Studies demonstrate that CD4^+^ T cells recognize a broader spectrum of latent EBV protein-derived epitopes and host-derived epitopes that are not recognized by CD8^+^ CTLs, implying non-redundant roles of CD4^+^ and CD8^+^ CTLs upon encountering EBV-infected cells [15,16,41,42].

Overall, despite different Eomes expression patterns in humans and mice, the killing activity of EBV-induced CD4^+^ CTLs was not inferior to that of CD8^+^ CTLs, pointing to T-bet as a conserved key transcriptional regulator in EBV-induced CD4^+^ and CD8^+^ T cells across species. The functional differences between cytotoxic CD4^+^ T cells and CD8^+^ T cells, as well as the clinical relevance of cytotoxic CD4^+^ T cells, should be determined in disease-specific contexts for immunotherapeutic strategies.

## 5. Conclusions

EBV-specific cytotoxic CD4^+^ T cells markedly express T-bet, Granzyme B, and Perforin, correlating with killing activity, which could reflect mechanisms shared with cytotoxic CD8^+^ T cells. T-bet-mediated transcriptional regulation may explain the similarity of EBV-mediated cytotoxic effector T cell differentiation between CD4^+^ and CD8^+^ T cells, suggesting that cytotoxic T cell differentiation may be restricted by environmental input rather than T cell subset.

## Data Availability

The complete microarray data are available at the Gene Expression Omnibus (http://www.ncbi.nlm.nih.gov/projects/geo; accession number GSE206802).
